# Evaluation of an integrated 3D‐printed phantom for coronary CT angiography using iterative reconstruction algorithm

**DOI:** 10.1002/jmrs.387

**Published:** 2020-03-27

**Authors:** Kamarul A. Abdullah, Mark F. McEntee, Warren Reed, Peter L. Kench

**Affiliations:** ^1^ Faculty of Health Sciences Universiti Sultan Zainal Abidin Terengganu Malaysia; ^2^ Discipline of Medical Radiation Sciences Faculty of Health Sciences The University of Sydney Lidcombe New South Wales Australia

**Keywords:** reconstruction settings, coronary CTA, dose optimisation, phantom, image quality

## Abstract

**Introduction:**

3D‐printed imaging phantoms are now increasingly available and used for computed tomography (CT) dose optimisation study and image quality analysis. The aim of this study was to evaluate the integrated 3D‐printed cardiac insert phantom when evaluating iterative reconstruction (IR) algorithm in coronary CT angiography (CCTA) protocols.

**Methods:**

The 3D‐printed cardiac insert phantom was positioned into a chest phantom and scanned with a 16‐slice CT scanner. Acquisitions were performed with CCTA protocols using 120 kVp at four different tube currents, 300, 200, 100 and 50 mA (protocols A, B, C and D, respectively). The image data sets were reconstructed with a filtered back projection (FBP) and three different IR algorithm strengths. The image quality metrics of image noise, signal–noise ratio (SNR) and contrast–noise ratio (CNR) were calculated for each protocol.

**Results:**

Decrease in dose levels has significantly increased the image noise, compared to FBP of protocol A (*P* < 0.001). As a result, the SNR and CNR were significantly decreased (*P* < 0.001). For FBP, the highest noise with poor SNR and CNR was protocol D with 19.0 ± 1.6 HU, 18.9 ± 2.5 and 25.1 ± 3.6, respectively. For IR algorithm, the highest strength (AIDR3D_strong_) yielded the lowest noise with excellent SNR and CNR.

**Conclusions:**

The use of IR algorithm and increasing its strengths have reduced noise significantly and thus increased the SNR and CNR when compared to FBP. Therefore, this integrated 3D‐printed phantom approach could be used for dose optimisation study and image quality analysis in CCTA protocols.

## Graphical abstract

This article acknowledges the use of 3D‐printing to develop a phantom for CT dose optimisation study. A 3D‐printed cardiac insert phantom was positioned into an anthropomorphic chest phantom and scanned with a multi‐slice CT scanner. An objective image analysis was performed. In summary, this phantom could be used for dose optimisation study especially when evaluating iterative reconstruction (IR) algorithm.

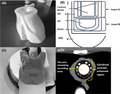

## Introduction

Coronary CT angiography (CCTA) has emerged as one of the most practical diagnostic imaging modality for investigating coronary artery disease. A minimal invasive procedure and less motion artefacts produce a reliable alternative to the invasive coronary angiography.^1^, ^2^ Recently, the number of CCTA scans being requested by the radiologists and cardiologists has increased drastically, mainly due to the improved spatial and temporal resolution.^3^ However, radiation dose associated with CCTA has raised serious concerns in the literature. The radiation dose carries a risk of developing malignancy to the patients.^4^, ^5^ Therefore, dose reduction strategies must be implemented to reduce the radiation dose as low as reasonably achievable.

Reconstruction algorithms are one of many strategies to reduce dose in CCTA. Currently, filtered back projection (FBP) is the mostly used image reconstruction algorithm. However, FBP results in images that can be deteriorated by both electronic and quantum noise.^6^ Iterative reconstruction (IR) algorithms allow using low‐dose CT protocols while maintaining the image quality. It uses statistical noise models to optimise the image quality of the final image.^7^, ^8^ This requires multiple steps, and with every step, noise is reduced according to the specific statistical model of the IR algorithms. IR algorithms can be represented by various strengths to determine the power of noise reduction. Therefore, CT image quality should be assessed to characterise the performance of IR algorithms.

Imaging phantoms are widely used as a tool for assessing the performance of IR algorithms for dose reduction in CCTA scans. Many phantoms, such as the Catphan^®^ (The Phantom Laboratory, Salem, NY) phantom, used in the previous studies^9^, ^10^, ^11^ to provide a good first‐order approximation of image quality. However, it is possible that such phantoms are not fully adequate to assess the impact of IR algorithms due to their current shape, the complexity of IR algorithms and the different types of patient body habitus, which can influence the radiation dose during CCTA scans. On the other hand, anthropomorphic phantom, such as the Lungman Phantom (Kyoto Kagaku, Japan), provides very similar shape to patients’ anatomy. Therefore, a combination of this phantom with three‐dimensional (3D)‐printed cardiac insert would be an appropriate simulation of heart scanning.

In our previous work,^12^ this 3D‐printed phantom has only been validated for Hounsfield unit (HU) values but it has not been validated for image noise, signal–noise ratio (SNR) and contrast–noise ratio (CNR). Therefore, in this current study, these characteristics of objective analysis will be used to evaluate the image quality especially for CCTA scans.

## Materials and methods

**Figure 1:**
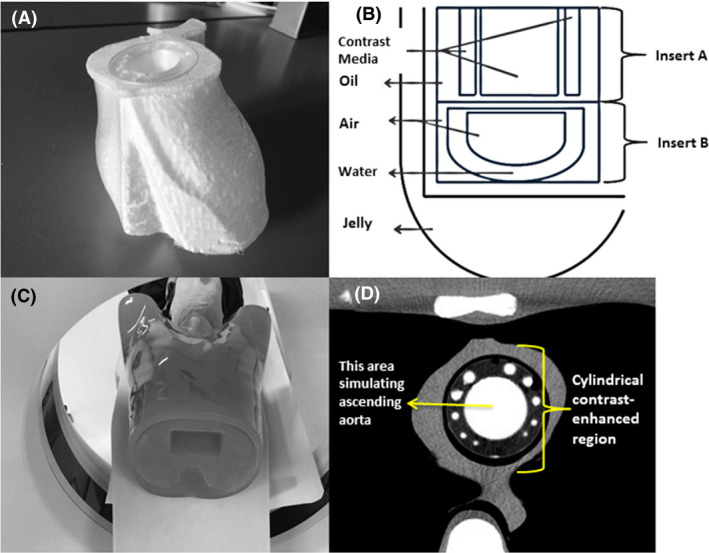
A, The 3D‐printed cardiac insert phantom. B, A schematic diagram of the phantom with all filled materials. C, The anthropomorphic chest phantom, containing the 3D‐printed cardiac insert phantom, is placed on the scanner couch. D, An axial CT image shows the contrast‐enhanced region of the 3D‐printed cardiac insert phantom; the centre simulates the contrast filled ascending aorta; and the varying size diameters of cylindrical demonstrate coronary arteries.

The 3D‐printed cardiac insert phantom is a similar shape and size to the cardiac insert of an anthropomorphic chest phantom (Lungman N‐01, Kyoto Kagaku Co., Ltd., Kyoto, Japan) and filled with different attenuating materials (Fig. 1A). The phantom’s filling materials were composed of a jelly (27.24 ± 2.67 HU), water (−6.83 ± 3.09 HU), oil (−93.73 ± 4.35 HU) and air (−996.77 ± 2.35 HU). Cylindrical structures simulating the coronary vessels and ascending aorta were filled with contrast media (354.33 ± 3.21 HU) (Fig. 1).

### Acquisition protocols

The 3D‐printed cardiac insert phantom was placed in the anthropomorphic chest phantom and scanned using a 16‐slice CT scanner (Alexion, Toshiba Medical Systems Co Ltd., Otowara, Japan) (Fig. 1C, D). The baseline exposure factors were based on the study centre’s standard CCTA protocols of 120 kVp and 300 mA (Protocol A). Additional acquisitions were made at 200 (Protocol B), 100 (Protocol C) and 50 mA (Protocol D). The volume of CT dose index (CTDI_vol_) for the 4 protocols was 19.2 mGy, 11.6 mGy, 5.8 mGy and 2.9 mGy, representing dose reductions of 40%, 70% and 85%, respectively. These dose reductions were chosen based on the previous work^6^. The detector collimation was 1 x 16 mm, the display field of view (DFOV) was 350 mm, and gantry rotation time was 0.75 s. Data acquisitions of the phantom were repeated thirty times for each exposure settings.

### Reconstruction settings

Protocol A was reconstructed only with the FBP, as the standard algorithm. Protocols B, C and D were reconstructed with the FBP and the IR algorithm of *adaptive iterative dose reduction three‐dimensional* (AIDR3D) (Toshiba Medical Systems Co Ltd., Otowara, Japan). The AIDR3D is the manufacturer’s commercial hybrid IR algorithm, which combines reconstruction in the raw data and image space. The iterations are executed in image space only, where the edge preservation and the smoothing are performed. The corrected image was blended with the initial image from the raw data to keep the noise granularity.^13^ The AIDR3D has three different strengths: mild, standard and strong. Table 1 shows the imaging parameters. Note: The IR algorithm is referred as the AIDR3D in the text and the figures.

**Table 1:** CT acquisition parameters and reconstruction settings.

Parameters				
Scanner type	Toshiba Alexion			
Detector collimation (mm)	16 × 1.0			
Field of view (mm)	160			
Helical Pitch (HP)	23			
Rotation time (s)	0.75			
Scan range (mm)	125			
Tube Voltage (kV)	120			
Tube current (mA) (protocol)	300 (A)	200 (B)	100 (C)	50 (D)
CTDI_vol_ (mGy) (protocol)	19.2 (A)	11.6 (B)	5.8 (C)	2.9 (D)
Reconstruction	FBP, AIDR3D mild, standard and strong			

Abbreviations: AIDR3D, adaptive iterative dose reduction three‐dimensional; FBP, filtered back projection.

### Image quality and dose reduction

A region of interest (ROI) was placed in the centre of contrast‐enhanced region that simulates the contrast filled ascending aorta for each slice of axial CT images. The size of ROI was adjusted to the maximum allowed area within that region. The measurements were made from 15‐slices at four dose levels, resulting in 15 × 4 = 60 slices. As the acquisitions were repeated thirty times, the total of images measured was 60 × 30 = 1,800 slices for each reconstruction. Image noise was quantified as standard deviation (SD) of attenuation values within the ROI. The SNR and CNR were calculated using equations 1 and 2, respectively.^14^, ^15^ The SNR was calculated by dividing the mean attenuation values (HU) by the corresponding SD (Equation 1).

(1) $$SNR=\frac{HU_{\mathrm{mean}}}{\mathrm{SD}}$$

The *CNR* was calculated as the difference between the two mean *HU* values (A and B) divided by the *SD* of the first material (A) (Equation 2). A pair of contrasts was measured (the contrast media (A) and the oil (B)) to simulate the ascending aorta and the fat.

(2) $$CNR=\frac{HU_{\mathrm{mean}}\left(A\right)-HU_{\mathrm{mean}}\left(B\right)}{SD\left(B\right)}$$

Data analyses were carried out using Statistical Package for the Social Science (SPSS, version 21; IBM Corp., New York, NY, USA). The image noise, SNR and CNR values were tested for normality by the Shapiro–Wilk test. Analysis of variances (ANOVA) test examined the differences between image noise, SNR and CNR.

## Results

**Figure 2:**
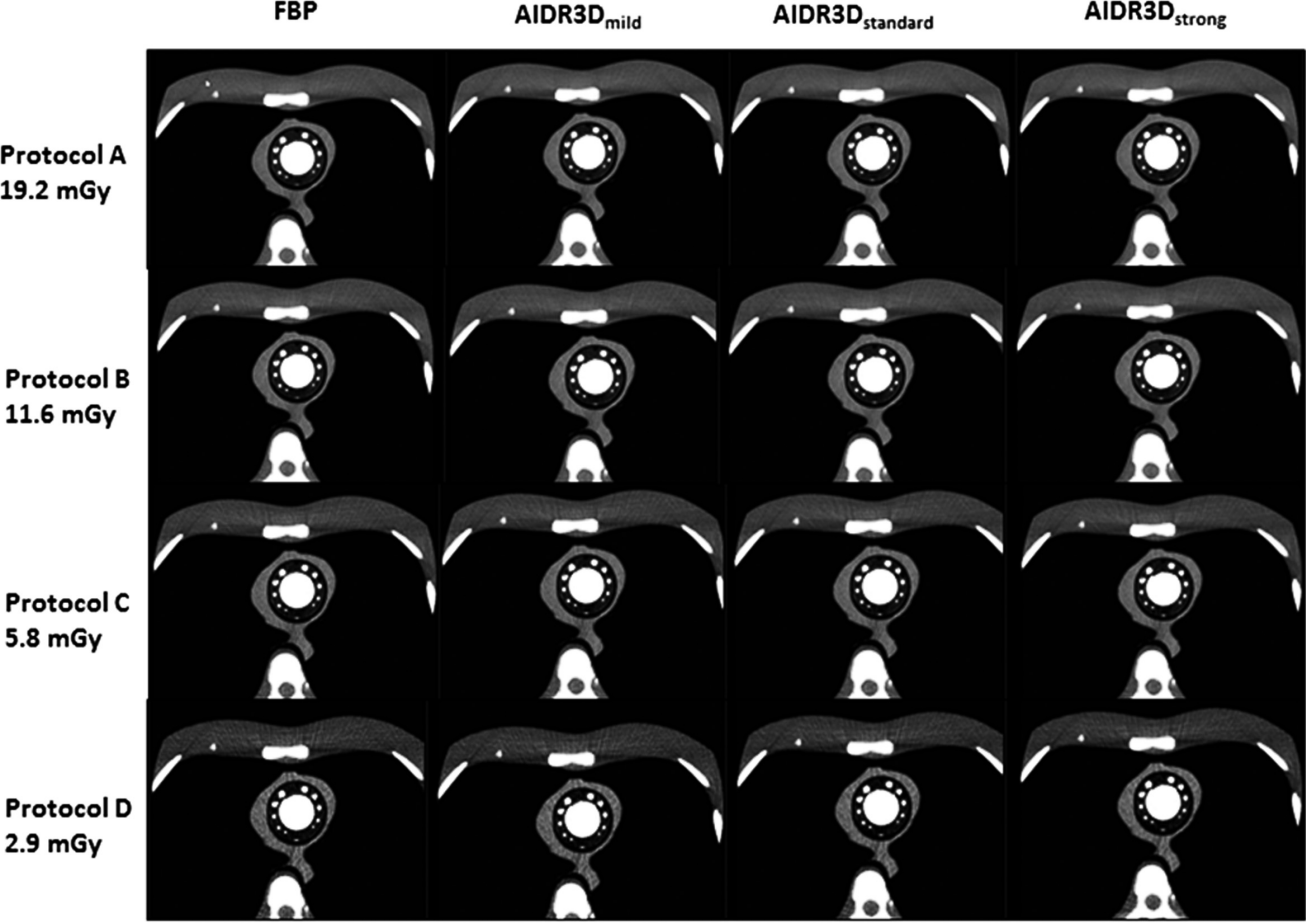
CT images of the 3D‐printed cardiac insert phantom at four dose levels in rows and reconstruction methods, FBP, AIDR3D_mild_, AIDR3D_standard_, and AIDR3D_strong_, in columns. The insert contains contrast‐material to simulate the ascending aorta and varying size of coronary arteries during cardiac CT imaging of CCTA. The images of Protocol D show the most clearly of noise pattern especially at the anterior region, as compared to the Protocols A, B, and C.

The resulting CT images of the associated 3D‐printed cardiac insert and the anthropomorphic chest phantom are shown in Figure 2.

### Image noise, SNR and CNR

Table 2 presents the image noise, SNR and CNR values. The FBP image noise (HU values) for protocols B, C and D were significantly higher than protocol A with the highest for protocol D (*P* < 0.001 for all). For protocols B, C and D, the AIDR3D_strong_ yielded the lowest image noise (9.8 ± 1.1, 12.4 ± 0.7 and 15.5 ± 1.2 HU, respectively) and the highest noise reduction (15%, 16% and 18%, respectively) when compared to the FBP. In contrast, the AIDR3D_mild_ showed the highest image noise (11.0 ± 1.2, 14.1 ± 0.9 and 18.0 ± 1.4, respectively) but the lowest noise reduction (<5%).

**Table 2:** Results of image noise, SNR and CNR at multiple dose levels using the 3D‐printed cardiac insert phantom.

Image reconstructions	19.2 mGy Protocol A	11.6 mGy Protocol B	Diff. (%)	5.8 mGy Protocol C	Diff. (%)	2.9 mGy Protocol D	Diff. (%)
Image noise (HU)							
FBP	9.5 ± 0.7	11.5 ± 1.2	21	14.7 ± 0.9	54	19.0 ± 1.6	99
AIDR3D_mild_		11.0 ± 1.2	15	14.1 ± 0.9	48	18.0 ± 1.4	89
AIDR3D_standard_		10.0 ± 1.1	6	13.0 ± 0.8	36	16.4 ± 1.2	72
AIDR3D_strong_		9.8 ± 1.1	3	12.4 ± 0.7	30	15.5 ± 1.2	63
SNR							
FBP	35.5 ± 3.4	31.2 ± 3.9	12	24.2 ± 2.8	32	18.9 ± 2.5	47
AIDR3D_mild_		31.5 ± 3.6	11	24.9 ± 2.8	30	19.7 ± 2.3	44
AIDR3D_standard_		33.9 ± 3.4	4	27.3 ± 3.2	23	21.5 ± 2.4	39
AIDR3D_strong_		35.0 ± 4.4	1	28.0 ± 2.8	21	22.2 ± 2.1	37
CNR							
FBP	46.6 ± 4.3	41.3 ± 5.7	12	32.0 ± 3.8	31	25.1 ± 3.6	46
AIDR3D_mild_		41.6 ± 5.2	11	33.0 ± 3.8	29	26.1 ± 3.3	44
AIDR3D_standard_		44.8 ± 4.9	4	36.0 ± 4.2	23	28.5 ± 3.3	39
AIDR3D_strong_		46.2 ± 6.3	1	37.0 ± 3.8	21	29.4 ± 3.0	37

Abbreviations: AIDR3D, adaptive iterative dose reduction three‐dimensional; FBP, filtered back projection.

Diff. (%): Represents the amount of noise reduction between the values of each protocol B, C or D, versus protocol A FBP.

The SNR values of protocol A was the highest when compared to the other three protocols. For FBP, the SNR was significantly reduced for protocols B, C and D, (12%‐47%) when compared to the protocol A (*P* < 0.001 for all). For protocols B, C and D, the AIDR3D_strong_ yielded the highest SNR while AIDR3D_mild_ showed the lowest compared to FBP and the highest SNR percentage differences (11%, 30% and 44%, respectively) when compared to AIDR3D_standard_ and AIDR3D_strong_.

The CNR values of AIDR3D in protocols B, C and D were significantly lower than the FBP in protocol A (*P* < 0.001 for all). Of these, the lowest CNR was measured in the protocol D. For FBP, the highest CNR was the protocol B (41.3 ± 5.7) with only 12% of percentage differences compared to the Protocol A. For IR algorithm, the higher strength of AIDR3D resulted in higher CNR. For protocols B, C and D, the AIDR3D_strong_ yielded the highest increase in CNR while AIDR3D_mild_ showed the lowest increase in CNR compared to the FBP for each protocol.

## Discussion

An organ‐specific 3D‐printed phantom, integrated with an anthropomorphic phantom, could be used for dose optimisation in CT examinations. In this study, the resulting images of an integrated 3D‐printed cardiac insert phantom and a chest phantom were analysed. The objective image analysis was performed to evaluate the IR algorithm at different strengths and dose levels in CCTA protocols.

The image noise, SNR and CNR are commonly used for the evaluation of IR algorithms.^16^ Although these objective noise characteristics of the CT image are just one metric of image quality, but it is likely that any changes made on them would affect the visualisation or measurement in a clinical task.^17^, ^18^, ^19^ For example, the visualisation of low contrast liver lesions is noise dependant, so reduction in noise would result in clearer visibility of the lesion. For CCTA, the image noise is usually measured in the centre of the ascending aorta as it is the area of the highest density of contrast‐enhanced region.^20^, ^21^, ^22^ In this study, the ROI was placed at the centre of the largest diameter of the contrast‐enhanced region to simulate the ascending aorta and measure the image noise, SNR and CNR. In line with previous studies,^15^, ^23^, ^24^, ^25^, ^26^, ^27^, ^28^ the results of 3D‐printed cardiac insert phantom images show that IR algorithm has significantly less image noise as compared to FBP. In addition, increasing the strength from AIDR3Dmild to AIDR3Dstrong has resulted in a range of noise reduction with improved measures of SNR and CNR.

The results of this phantom study could infer dose reduction potential if performed in clinical settings. This is especially indicated by the image quality of IR algorithm at the reduced dose of 11.6 mGy (CTDIvol), which was similar to FBP at 19.2 mGy (CTDIvol). The similar results may be seen in paediatric patients. The integrated 3D‐printed phantom and chest phantom can be reproduced to allow smaller heart size and chest region. As such, the results would aid in development of dose optimised protocols for a department and thus reducing the risks associated with the radiation received by all types of patients. This opens up the potential of creating size‐specific phantoms, normal variant‐specific phantoms and pathology‐specific phantoms for optimisation. Researchers could use this 3D printing methodology to investigate its effects on radiation dose of rare normal variants such as situs inversus or dextra cardia.

Our results using the integrated 3D‐printed cardiac insert phantom suffer several limitations. First, the image quality metrics were objective measures of noise only. However, in real patients, the subjective measurements of image quality are also important for CT images with lesions. We aim to include subjective measurements in future studies using cylindrical contrast‐enhanced region of varying diameters to simulate lesion detectability. Second, the 3D‐printed cardiac insert involves no physiological motion such as breathing, heartbeat or peristalsis. We recognised that motion contributes a significant impact on the image quality; however, the aim was to investigate the influence of post‐acquisition factor of image reconstruction. As such, the motion factor was excluded. Also, the ECG‐gating method used in the recent studies^29^, ^30^, ^31^ has shown that images used for reconstruction are effectively still during the acquisitions. Further work plans to improve this design by introducing features to simulate the heart movement during a cardiac cycle. Third, the scanning was performed with the use of 16‐slice CT scanner and single type of IR algorithm. Therefore, the results may not apply to more advanced CT scanners and different IR algorithms. However, in the future work, this study will be conducted using 64‐slice CT scanners or above and multiple types of IR algorithm for comparison. Last, the physical geometry of our 3D‐printed cardiac insert phantom is not a patient‐specific model. The phantom has smooth surface with less complexity than in the real heart. These cardiac phantom features could produce different measurement of image quality metrics as shape and size may affect the image noise. However, in future study, the work will focus on developing patient‐specific cardiac insert to allow personalised 3D‐printed models.

In summary, using IR algorithm and increasing its strengths have reduced noise significantly and thus increased the SNR and CNR when compared to FBP. Therefore, this integrated 3D‐printed cardiac insert and chest phantoms approach has enabled image quality analysis and could be used for dose optimisation in CCTA protocols.

## Ethics

No human or animal testing is required for this study.
